# Cardiac Xenotransplantation: Progress in Preclinical Models and Prospects for Clinical Translation

**DOI:** 10.3389/ti.2022.10171

**Published:** 2022-03-23

**Authors:** Avneesh K. Singh, Corbin E. Goerlich, Aakash M. Shah, Tianshu Zhang, Ivan Tatarov, David Ayares, Keith A. Horvath, Muhammad M. Mohiuddin

**Affiliations:** ^1^ Department of Surgery, School of Medicine, University of Maryland, Baltimore, MD, United States; ^2^ Revivicor Inc., Blacksburg, VA, United States; ^3^ National Heart, Lung, and Blood Institute, National Institute of Health, Bethesda, MD, United States

**Keywords:** xenotransplantation, cardiac transplantation, transplantation, pre clinical model of xenotransplantation, heart transplantation

## Abstract

Survival of pig cardiac xenografts in a non-human primate (NHP) model has improved significantly over the last 4 years with the introduction of costimulation blockade based immunosuppression (IS) and genetically engineered (GE) pig donors. The longest survival of a cardiac xenograft in the heterotopic (HHTx) position was almost 3 years and only rejected when IS was stopped. Recent reports of cardiac xenograft survival in a life-sustaining orthotopic (OHTx) position for 6 months is a significant step forward. Despite these achievements, there are still several barriers to the clinical success of xenotransplantation (XTx). This includes the possible transmission of porcine pathogens with pig donors and continued xenograft growth after XTx. Both these concerns, and issues with additional incompatibilities, have been addressed recently with the genetic modification of pigs. This review discusses the spectrum of issues related to cardiac xenotransplantation, recent progress in preclinical models, and its feasibility for clinical translation.

## Introduction

Xenotransplantation (XTx) is an alternative source of a human organ for patients with end-stage organ failure. Many of these patients will die waiting for a human organ, as the current availability of donor organs falls short of its demand. In the past few years, substantial progress has been made in the xenotransplantation field. With the discovery and use of novel molecular biology techniques, genetically engineered (GE) porcine organ donors have been created to overcome numerous XTx barriers. The first transgenic pig for XTx was produced expressing human complement regulatory protein (hCRP) decay acceleration factor (hDAF). Organs from these pigs were transplanted in non-human primate (NHP), but hyperacute rejection (HAR) was only partially avoided (1, 2), and antibody-mediated immune response induced to terminal galactose sugar molecules (α1-3 Galactose or Gal) expressed on graft vascular endothelial cells continued to cause HAR. By using gene-editing techniques, Gal antigen was knocked out in pigs, and organs from these pigs were protected from HAR (3–5).

**FIGURE 1:**
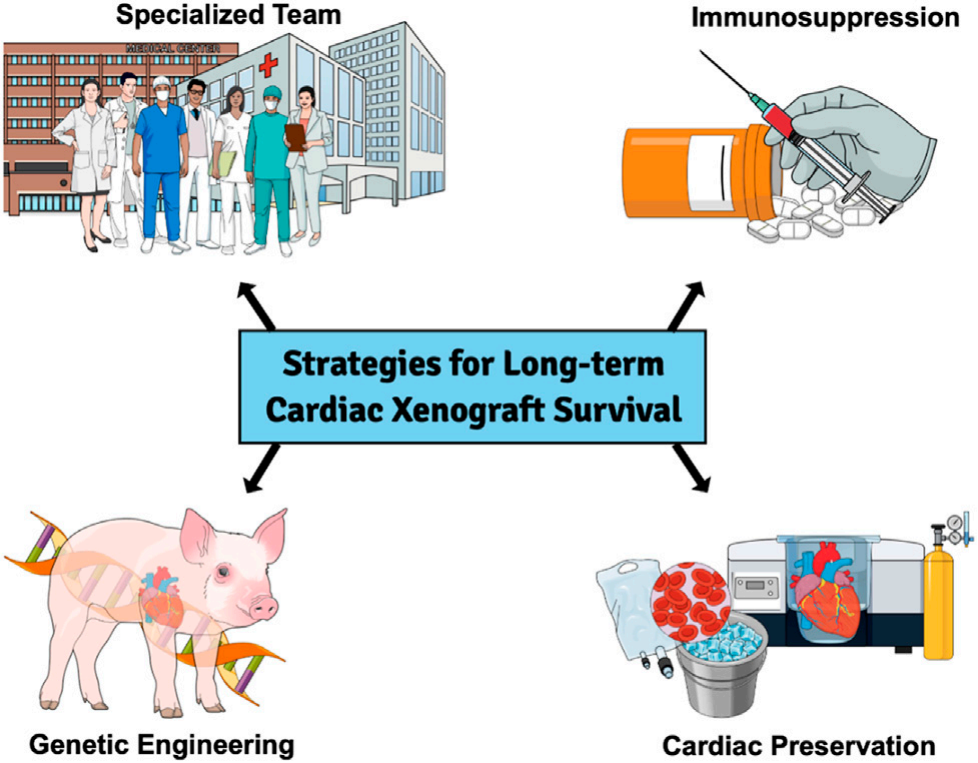
Strategies for achieving success for long term cardiac xenograft survival.

Other combinations of antigen knockout and human transgene expressing GE pigs were produced, and xenograft survival was extended further (6–13). We (HHTx Heart) and others (Kidney, Liver) have also reported long-term xenograft survival in NHP from genetically modified pigs (10, 12, 14–17). Recently, Langin et al. reported consistent survival in an experimental life-supporting (OHTx) in NHP (18). Strategies which have helped to achieve this success have also been summarized in Figure 1. In this review, we discuss the challenges faced in cardiac xenotransplantation and solutions that have culminated from the last several decades of work and speculate on the next steps required to make cardiac XTx a clinical reality (19, 20).

## Challenges for Cardiac Xenotransplantation

### Immunological

#### Preformed Natural and Elicited Antibodies

The presence of natural preformed antibodies (nAbs) against pig antigens in recipients is a primary and significant hurdle for the success of cardiac XTx. These antibodies trigger immune responses and causes hyperacute (HAR) and acute humoral xenograft rejection (AHXR) (21). These nAbs against donor antigens (xenoantigens) trigger the activation of complement proteins, which further cause activation and damage to endothelial cells, leading to platelet aggregation and microvascular thrombosis. This ischemic injury leads to the destruction of cardiomyocytes, interstitial hemorrhage, and eventually fibrosis. Most of nAbs are against porcine carbohydrate antigens not found in humans and NHP. The most predominant of these is Galactose-α1-3 galactose, due to the acquired mutation of α1-3 galactosyltransferase (GT), an enzyme responsible for synthesizing this carbohydrate antigen. Others include SDa, and N-glycolylneuraminic acid (Neu5Gc). While preformed antibody responses dominate Gal antigens, it has been shown that elicited Abs responses can occur in cardiac XTx also towards these other antigens (i.e., non-Gal antigens) (22, 23, 24–30). Elicited Abs also play a major role in posttransplant thrombotic microangiopathy (TM), consumptive coagulopathy (CC), and AHXR (10, 31–34).

#### Cellular Xenograft Rejection

Besides HAR and AHXR, acute cellular rejection of cardiac xenografts can be mediated by innate (i.e., macrophages, neutrophils, dendritic cells, and NK cells) and adaptive (i.e., T and B cells) immune responses (35–37). However, acute CXR has not been reported frequently in xenotransplantation (34, 38). Innate immune cells, like macrophages and NK cells, have been found in pig organs perfused with human blood *ex vivo* and in pig-to-NHP xenografts, which may trigger CXR (34). Macrophages may also be activated by xenoreactive T cells and release proinflammatory cytokines (e.g., tumor necrosis factor-alpha (TNF-α, IL-1, and IL-6), which can further stimulate T cells. Both macrophages and NK cells can also be activated by direct interaction between donor endothelial antigens and their surface receptors, which may trigger CXR by direct NK cytotoxicity or antibody-dependent cellular cytotoxicity (ADCC) (39, 40).

T cells can be activated through both direct and indirect pathways after xenotransplantation. However, the responses against xenoantigens, especially indirect responses, are more robust than seen in allotransplantation (41). T cell activation requires interaction between TCR and MHC peptide complex from the antigen-presenting cells (APC) and a costimulatory signal (e.g., CD40–CD154 and CD28–CD80/86 pathway interactions) (42, 43).

### Coagulation Dysfunction

Coagulation dysregulation is also another major impediment to the success of xenotransplantation. The most extreme manifestations of it are systemic consumptive coagulopathy, characterized by thrombocytopenia and bleeding, which ultimately leads to graft loss due to ischemia from thrombotic microangiopathy (TM). Coagulation is a complex pathway that involves interactions of inflammation, vascular injury, heightened innate, humoral, and cellular immune responses. Incompatibilities between primate and pig coagulation/anti-coagulation factors can alter their function, contributing to coagulation dysfunction (44, 46). Notable proteins with cross-species incompatibilities include tissue factor pathway inhibitor (TFPI), thrombin, thrombomodulin (TBM), endothelial protein C receptor (EPCR) and CD39 (45–47).

Complement is also able to activate the clotting cascade, as it can be activated by the binding of complement fixing antibodies onto endothelium. As an example, activated product of complement C5a has been reported to induce tissue factor (TF) activity in endothelial cells (48) and has been reported to modifying the balance between pro- and anti-coagulation (49).

Preformed and elicited antibodies promote coagulation by activating porcine endothelial cells and platelets and contribute to graft loss due to TM (50–52). Systemic inflammatory responses and proinflammatory cytokines (notably IL-6) also upregulate or recruit recipient tissue factors (TF) on platelets and monocytes by interacting with porcine vascular endothelial cells which can lead to coagulation through thrombin production (54, 55).

### Viral Transmission

A potential problem for cardiac xenotransplantation is a zoonotic viral transmission from swine. Most notable of which is a porcine endogenous retrovirus (PERV). There is no report yet for *in-vivo* pig-to-human PERVs transmission so the true risk in the context of xenotransplantation is not known (56). But, *in-vitro* studies have shown that PERVs could be transmitted from pig cells to human cells (57). Provirus DNAs of PERVs can be genetically transferred to offspring and cannot be eliminated by specified pathogen-free (SPF) breeding. Like other retrovirus, PERV theoretically predispose to the risks of tumors, leukemia, and neurodegeneration (58). However, studies have shown complete elimination of all copies of PERV in donor pigs (57).)

Porcine circovirus (PCV) from the Circoviridae family is also highly distributed among pigs and wild boars. Previously, two types of PCV1 and PCV2 have been characterized (59). PCV1, which is isolated from pig kidney cell culture (PK15 cells), and recently, Liu et al. have demonstrated that PCV2 can infect human cells *in vitro* with a reduced infection efficiency compared to pig PK-15 cells. Kruger et al. were unable to identify PCV1 and PCV2 in GE pigs. However, two other subtypes PCV3a and PCV3b, were found in the spleen, liver, lung kidney, and explanted heart of recipient baboons of GE cardiac xenografts after OHTx (60). The presence of PCV3 in the OHTx recipient baboon was higher among long-term survivors. However, the significance of PCV in causing clinical disease is unknown.

### Xenograft Growth

Although there are several anatomical and physiological similarities between pigs and humans (or NHPs), their organs’ growth rate is significantly different (61). Therefore, the use of minipigs has been suggested as their mature growth rate is 1/3 that of wild type Yorkshire (domestic) pigs (62). However, mostly domestic pigs have been used even for genetic modifications, but organs from these GE pigs continue to grow too large (61). Therefore, juvenile GE pigs are being preferred, but even still, continued organ growth after transplantation has been reported (8, 62, 63). Längin et al. have also reported left ventricular hypertrophy after pig OHTx in NHPs, but it is unclear its origin and whether this is from rejection, physiologic mismatch or natural growth (i.e., intrinsic or extrinsic causes or a combination of both) (18). In contrast, others have not seen pig heart growth after HHTx until the xenograft underwent delayed xenograft rejection (14, 15). In these experiments heart graft size was maintained until the co stimulation pathway blockade was reversed by stopping the anti-CD40 antibody.

While the growth of other organs such as the kidney can be accommodated within the abdomen, the growth of a heart xenograft could be problematic due to its position in the non-compliant chest and must be addressed before clinical translation.

## Overcoming the Challenges for Successful Cardiac Xenotransplantation

### Generation of Genetically Modified Donors

Several genetic strategies have been developed to prevent early graft failure from preformed antibodies and coagulation dysfunction resulting in generation of GE pigs. Genome editing using zinc-finger nucleases, transcription activator-like effector nucleases, or CRISPR-Cas9 is being used to delete multiple genes with high precision to produce GE pigs. Several pig genes are knocked out (e.g., α1-3 galactosyltransferase, B4GALNT2 and CMAH) and human genes are overexpressed (e.g., hCD46, hTBM, hEPCR, hTFPI, hCD39, etc.) in these GE pigs (Table 1).

**TABLE 1:** The “genetic toolbox” central to our strategies to minimize or abolish hyper-acute and delayed humoral rejection.

Genetic modification	Mechanisms	Properties
Alpha-Gal KO (GTKO)	Deletion of immunogenic Gal antigen expression	Anti-Immunogenic
B4GalNT2 KO	Deletion of B4Gal	
CMAH KO	Deletion of Neu5Gc	
hHO-1	Decreases oxidative products	Anti-Apoptotic
hHLA-E	Protects the graft against human killer cells	Anti-Inflammatory
hCD46	Suppresses human complement activity	
hCD55 (DAF)	Suppress human complement activity	
hEPCR	Activates Protein C	Anti-Coagulation
hTFPI	Inhibits Factor Xa	
hvWF	Reduces platelet sequestration and activation	
hTBM	Binds human thrombin, and activates Protein C via activated thrombin	
Multi-Genetic Modified Pigs		
• GTKO.hCD46		
• GTKO.CD55(DAF) (64, 65)		
• GTKO.hCD46.CD55(DAF) (14)		
• GTKO.hCD46.hTBM (15, 18, 63)		
• GTKO.hCD46.CD55.EPCR.TFPI.CD47 (63)		
• GTKO.hCD46.hTBM.CD47.EPCR.HO1		
• GTKO. B4GalNT2KO (66)		
• GTKO. B4GalNT2KO.hCD46.hHLAE		
• GTKO.B4KO.hCD46.hTBM.hEPCR. hCD47.hHO1.hVWF		
• GTKO.CMAHKO (67)		
• GTKO. B4GalNT2KO CMAHKO (68)		
• GTKO.CMAHKO.hCD46.hCD47. hTFPI		
• GTKO.CMAHKO.hCD46.hEPCR. hDAF		
• GTKO.CMAHKO.hCD46.hEPCR. hDAF.hTBM. hHO1		
• GTKO.CMAHKO.B4GalNT2KO.hCD46.hDAF		
• GTKO.B4GalNT2KO.GHRKO. hCD46.hTBM.hEPCR.hCD47 (69)		
• GTKO.B4GalNT2KO.CMAHKO.GHRKO. hCD46.hTBM.hEPCR.DAF.hCD47.HO1 (69)		

CMAH, cytidine monophospho-N-acetylneuraminic acid hydroxylase; EPCR, Endothelial Protein C Receptor; HO-1: Heme Oxygenase -1; TFPI, tissue factor pathway inhibitor; HLA, human leukocyte antigen; h, human; vWF, von Willebrand Factor; TBM, thrombomodulin.

The genetic constructs listed in Table 1, and the GE pigs produced, have been tested to various degrees. Kuwaki et al. reported the longest (179 days) heterotopic cardiac xenograft survival of GTKO hearts in NHP (6). Chen et al. also found an advantage in using GTKO pig kidneys over previously used transgenic kidneys (5). Recently, GTKO pigs along with other transgene have significantly improved the cardiac xenograft survivals in NHP to months in OHTx and years in HHTx models (14, 15, 69, 70).

CRISPR technology has now come into vogue as it affords complex genetic constructs to be employed with the highest fidelity compared to other techniques. Two carbohydrate antigen-expressing genes (e.g., GT and CMAH) have been deleted, and “double knockouts” (GTKO.CMAHKO) have been constructed (71, 72). Burlak et al. reported a reduced binding of human antibodies to cells from these GTKO. CMAH KO pigs (67). Later, Tector’s group has produced three carbohydrate antigen knockout (TKO) pigs (i.e., GTKO.CMAHKO.B4GALNT2KO), which included deletion of B4GALNT2 responsible for SDa antigen along with GT and CMAH genes (26, 31, 73). They demonstrated that the binding of human IgG and IgM antibodies to peripheral blood mononuclear cells and red blood cells from triple knockout pigs was significantly reduced. Niu et al. inactivated all known porcine endogenous retrovirus (PERVs) within pig xenograft donors (74). A combination of various genetic constructs is being developed by other groups as well, a testament to the technology’s ability to move the field forward quickly. “Multi-gene” expressing cardiac xenografts’ effect on overall graft function and survival in HHTx and OHTx is currently a topic of investigation in our lab and others.

### Immunosuppression

To achieve long-term xenograft survival, various immunosuppressive (IS) drug regimens have been used along with GE pigs. Earlier conventional corticosteroids and calcineurin based (CSA) immunosuppression (IS) was used in NHP recipients, which prevented acute rejection, but failed to prolong cardiac xenograft survival (75–77). The longest reported cardiac xenograft survival using a CSA-based IS regimen was 32 days from a wild type (WT) pig (75) but was extended up to 99 days (median 26 days) using hDAF transgenic hearts (78). Various other IS regimens were used which include splenectomy or total body irradiation, non-antigenic alpha-Gal polyethylene glycol polymer (TPC) alone or in combination (9, 23, 79). Effect of these immunosuppression regimen on cardiac xenograft survival has been summarized in Table 2. Later, anti-thymocyte globulin (ATG), rituximab, mycophenolate mofetil, tacrolimus, and sirolimus were also used in various combinations as alternative regimens (10, 80–82). For complement inhibition, either cobra venom factor (CVF) or overexpression of complement regulatory protein gene expression for a donor organ or both were used (10, 15, 81). By using these IS drugs, McGregor et al. 2005 reported consistent graft survival (median 96 days; range, 15–137 days) in an HHTx model, but xenograft rejection was associated with a rise in non-Gal antibody titers. They did not observe a significant difference in graft survival when GTKO or GTKO.hCRP donors were used (35, 64).

**TABLE 2:** Progress in Cardiac Xenograft Survival (Heterotopic and Life Supporting Orthotopic) and Immunosuppression Regimen used.

Type of graft	Broad immunosuppression category	GE cardiac xenograft survival (Days)	References
Heterotopic		<1	(10)
	Without Immunosuppression		
	With Immunosuppression		
	• Without Corticosteroids^a^	3–62	
	• Total body irradiation^a^	8–15	(9)
	• Immunoadsorption^a^	9–39	(32, 83)
	• Thymic irradiation^a^	8–15	(84)
	• Splenectomy^a^	0–139	(84–87)
	• Immunosuppressive Reagents e.g., Cyclosporine, MMF 15-Desocyspergualin TPC, Gas914,Tacrolimus, Rapamycin^a^	0–139	(64, 76, 78, 82, 84, 91)
	• CVF^a^	16–179	(10, 14, 15, 81, 92, 93)
	• ATG^a^	5–236	(10, 14, 15, 86)
	• Anti-CD20^a^	0–236	(10, 14, 15, 86)
	• Costimulation blockade (Anti CD154 and anti CD40 Antibody)^a^	8–945	(10, 14,15, 81, 92, 93)
Orthotopic			
	With Immunosuppression		
	• Immunoadsorption, TBI, CsA, Methotrexate^a^	18–19	(79)
	• Immunosuppressive reagents, e.g., Cyclosporine, Cyclophosphamide, MMF, Tacrolimus, Rapamycin^a^	1–25	(75, 94–96)
	• CVF, ATG, Anti CD20, Anti-CD40 antibody, Non-ischemic preservation technique^a^	51–264	(18, 23, 69, 97)

a Introduction of new agents along with other immunosuppressive drugs.

Significant progress in cardiac XTx occurred when newer agents were used that block the co-stimulation, which aids in T cell activation upon antigen exposure (10, 11, 15, 92). In 2000, Buhler et al. demonstrated that the blocking of the CD40/CD154 pathway by anti-CD154 antibody prevents an induced anti-pig humoral response (99). Kuwaki et al. also reported the longest cardiac xenograft survivals for 179 days (median 78 days) (6) in HHTx with anti-CD154 antibody treatment. We have also reported more than 8-month survival of GTKO.CD46 cardiac xenograft in HHTx with continuous co-stimulation blockade by anti-CD154 antibody (25 mg/kg; clone 5C8) and B cell depletion with Rituxan at the time of transplantation (10). Although the use of anti-CD154 antibody has improved survival, it has been reported that anti-CD154 antibody is associated with bleeding and thrombotic complications such as consumptive thrombocytopenia and venous and arterial thrombi (10, 81, 99). As a result, replacement with an anti-CD40 (25 mg/kg; clone 2C10) monoclonal antibody (mAb), which targets the same interaction, has been the focus of the active investigation. When we used this antibody, there was no significant difference found in median 70 vs. 75 days) compared to anti-CD154 blockade.

However, we demonstrated that cardiac xenograft (GTKO.CD46. TBM) survival in HHTx was significantly prolonged (median 298 days) when the anti-CD40 antibody was used at a higher dose (50 mg/kg) (15, 100). Iwase et al. also demonstrated anti-CD40 mAb combined with belatacept proved effective in preventing a T cell response (14). The anti-CD40 mAb used in these studies is a mouse/rhesus chimeric IgG4 antibody, which may not be suitable for use in humans. Still, several other humanized anti-CD40 blocking antibodies under development can be used for human use if approved as an immunosuppression adjunct in cardiac XTx (101).

### Prevention of Viral Transmission

The risk of PERV transmission can be minimized by selecting PERV negative porcine donors. Thorough screening of PERV can be done by serology, western blot, ELISA, immunofluorescence, scanning electron microscopy, and PCR. Recently, Yang et al. have inactivated all PERV proviruses (62 copies of PERV’s gene *pol*, leading to a 1,000 times reduction in the virus’s ability to infect human cells) in the pig genome the CRISPR/Cas technique (102). The use of PERV inactivated pigs may provide tissue, organs that may address the safety issue from a porcine virus in pig-to-human xenotransplantation. However, the impact of PERV inactivation and gene editing on PERV-inactivated pigs and the necessity of these complex constructs is not known.

### Prevention of Xenograft Growth

In one approach, xenograft growth is controlled by using drugs such as rapamycin (8, 14, 18). Inhibition of mTOR protein kinase has been shown to control cell growth and proliferation to treat cancers in the clinical setting (103). Längin et al have used mTOR inhibitor and anti hypertensive drugs to control the blood pressures to prevent overgrowth cardiac xenograft in OHTx (18). Recently, Hinrichs et al. have produced GHRKO pigs in order to address intrinsic organ growth. They demonstrate that GHRKO pigs have slow or reduced growth, including their organs’ growth, compared to normal wild-type pigs (61, 104–107). Recently, Goerlich, et al. have examined intrinsic and extrinsic causes of graft growth after transplantation in an OHTx model using “multi-gene” pigs growth hormone receptor knockout pigs (GHRKO) (69). Post-transplantation xenograft growth was measured by echocardiography longitudinally after transplantation between multi-gene cardiac xenografts with and without GHRKO. Extrinsic causes of graft growth, namely blood pressure and heart rate, were left without treatment. GHRKO grafts demonstrated a 50.4% increase in LV mass up to 9 months (264 days) after OHTx compared to 140.1% in xenografts with a limited survival of less than 3 months. Terminal histology demonstrated fibrosis, interstitial edema and hemorrhage as the cause of this growth and not classical hypertrophy. Moreover, blood pressures and heart rates were significantly elevated after transplantation regardless of GHRKO status, suggesting physiologic mismatch occurs after transplantation. Altogether, these data suggest that post-transplantation xenograft growth in OHTx is multifactorial; largely driven by intrinsic growth with some extrinsic component not related to physiologic mismatch. Terminal histology would suggest this extrinsic component could be rejection related.

## Progress in Cardiac Xenotransplantation Toward Clinical Translation

**FIGURE 2:**
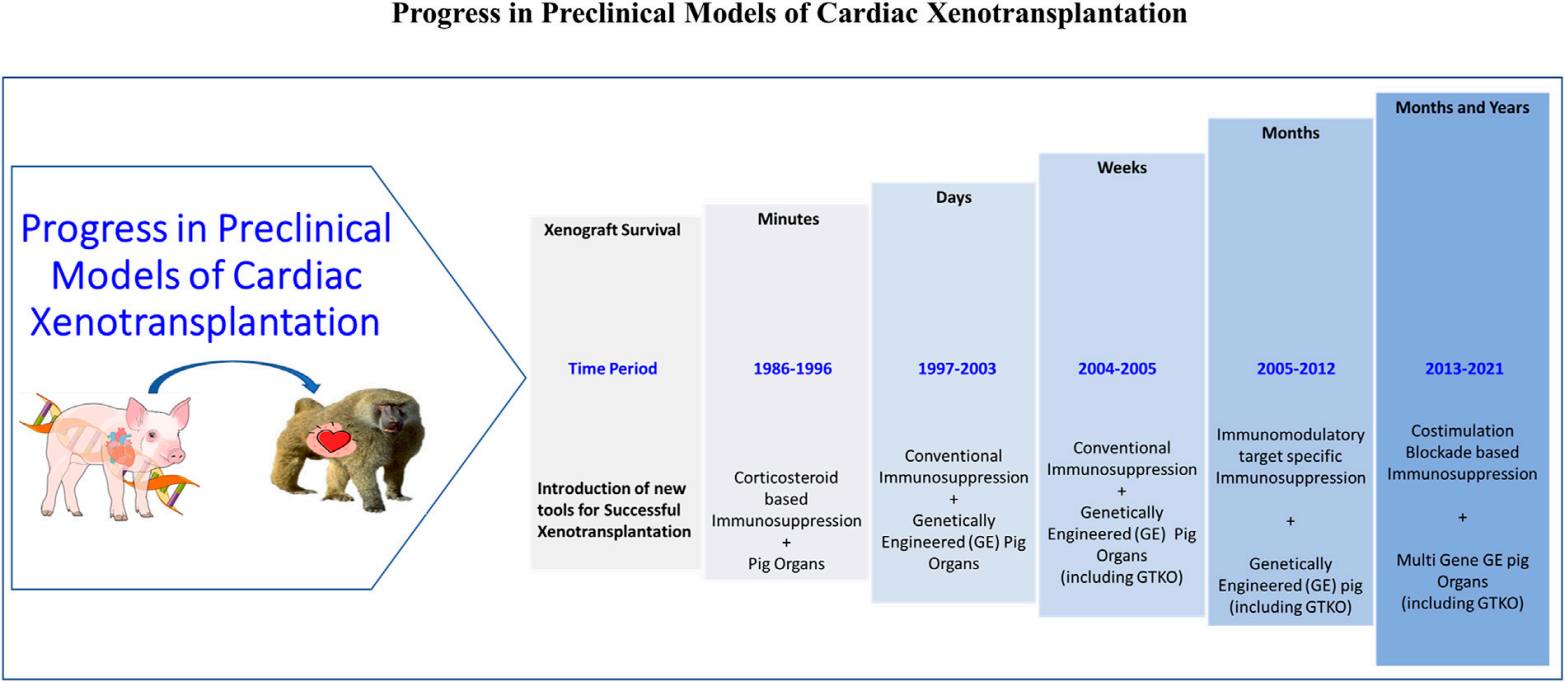
Timeline showing the progress in pre-clinical model of cardiac xenotransplantation.

The progress of cardiac xenotransplantation has been immense (Figure 2) but the transition from HHTx to OHTx (i.e., to the life-supporting function of xenografts) has been fraught with its own challenges as the recipient’s native heart is replaced entirely by the xenograft (108–112). Thus, any perturbations in the graft (arrhythmias, ventricular function, or rejection) can have devastating consequences to the recipient. Peri operative cardiac xenograft dysfunction (PCXD) has been observed in 40%–60% of OHTx which has also made the transition difficult (23). However, there has been a success in the OHTx with GTKO.hCD46.hTBM (3-GE) graft survival up to 6 months, despite these hurdles with the aid of non-ischemic continuous xenograft preservation (70, 112). This has been observed by others as well, but the underlying mechanism in cardiac preservation preventing primary graft dysfunction in this setting is poorly understood (113).

The advancement in donor genetic engineering capabilities has also resulted in xenografts with additional transgenes and knockouts for successful long-term OHTx survival. While multi-gene xenografts have certainly fallen into favor, there has been a recent increase in interest for “triple knock out (TKO)” xenografts, which lacks three carbohydrate antigens. In addition to Gal antigens, knockout for additional non-Gal antigens addresses other preformed antibodies that can contribute to humoral rejection. However, like our HHTx experience, we have also seen that hTBM is important in increased survival in xenografts, but specifically, we have seen that TKO grafts exhibit accelerated antibody-mediated rejection and increased incidence of thrombotic complications (16). This could be because of the lack of human transgenes in these TKO xenografts or because TKO xenografts create *de novo* synthesis, a novel xenoantigen on their surface due to CMAH knockout in the TKO pig that baboon recipients see as foreign (114).

However, multi-gene pigs with double and triple carbohydrate knockouts have been developed for cardiac xenotransplantation and are currently being tested in OHTx and HHTx models. Recently, we have achieved up to 264 day survival of a multi-gene cardiac xenograft with additional human transgene and knockouts (69). Notable modifications in these pigs were are carbohydrate enzyme KO (GTKO and β4GalNT2), growth hormone receptor knockout (GHRKO) and the addition of human transgenes (hCD46, hTBM, hEPCR and hCD47). We are testing cardiac xenograft survival which have over expression of other human genes (8–10 GE) in addition to these from pigs in OHTx with mixed success. These studies, along with others, will soon shed light on the advantages and disadvantages of iterative genetic modifications and pave the way for pre-clinical efficacy required for human clinical trials.

### Conclusion

We are now entering an exciting time in xenotransplantation with the progression of survival in preclinical models of pig cardiac xenotransplantation. With the understanding now that a multi-pronged approach toward these recipients’ immunosuppression increases graft survival, most critical of which to date is co-stimulation blockade, attempts to reduce the burden of immunosuppression has placed genetic engineering of cardiac xenografts in the forefront. Increasing the immunocompatibility of xenografts from genetically engineered pigs are a noble approach utilizing technology that has progressed the field further. However, genetic engineering should proceed with caution, utilizing *in vitro* evidence for every iterative improvement in the genetically engineered cardiac xenograft. Given the field’s current progression and demonstration of success, it is our opinion that multi-gene xenografts which include iterative addition of human transgenes or knockouts of pig genes along with targeted immunosuppression will pave the wave for clinical translation a reality.

## Abbreviations

B4GALNT2, beta-1,4-N-acetyl-galactosaminyltransferase; CC, consumptive coagulopathy; CMAH, CMP-N-acetylneuraminic acid hydroxylase; CRISPR, clustered regularly interspaced short palindromic repeats; CSA, cyclosporin; GHRKO, growth hormone receptor knockout; GTKO, 1-3 alpha Galactosyltransferase knockout; hCRP, human Complement Regulatory Protein; hDAF, human Decay Acceleration Factor; HHTx, Heterotopic Heart xenotransplantation; Neu5Gc, N-glycolylneuraminic acid; OHTx, Orthotopic Heart xenotransplantation; SPF, specified pathogen-free; TBM, thrombomodulin; TKO, triple knockout; TPC, alpha-Gal Polyethylene glycol polymer.
